# Monodisperse Picoliter Droplets for Low-Bias and Contamination-Free Reactions in Single-Cell Whole Genome Amplification

**DOI:** 10.1371/journal.pone.0138733

**Published:** 2015-09-21

**Authors:** Yohei Nishikawa, Masahito Hosokawa, Toru Maruyama, Keisuke Yamagishi, Tetsushi Mori, Haruko Takeyama

**Affiliations:** 1 Department of Life Science and Medical Bioscience, Waseda University, 2–2 Wakamatsu-cho, Shinjuku-ku, Tokyo 162–8480, Japan; 2 Research Organization for Nano & Life Innovation, Waseda University, 513, Wasedatsurumaki-cho, Shinjuku-ku, Tokyo 162–0041, Japan; 3 Core Research for Evolutionary Science and Technology (CREST), Japan Science and Technology Agency (JST), 5, Sanbancho, Chiyoda-ku, Tokyo 102–0075, Japan; National Cheng Kung University, TAIWAN

## Abstract

Whole genome amplification (WGA) is essential for obtaining genome sequences from single bacterial cells because the quantity of template DNA contained in a single cell is very low. Multiple displacement amplification (MDA), using Phi29 DNA polymerase and random primers, is the most widely used method for single-cell WGA. However, single-cell MDA usually results in uneven genome coverage because of amplification bias, background amplification of contaminating DNA, and formation of chimeras by linking of non-contiguous chromosomal regions. Here, we present a novel MDA method, termed droplet MDA, that minimizes amplification bias and amplification of contaminants by using picoliter-sized droplets for compartmentalized WGA reactions. Extracted DNA fragments from a lysed cell in MDA mixture are divided into 10^5^ droplets (67 pL) within minutes via flow through simple microfluidic channels. Compartmentalized genome fragments can be individually amplified in these droplets without the risk of encounter with reagent-borne or environmental contaminants. Following quality assessment of WGA products from single *Escherichia coli* cells, we showed that droplet MDA minimized unexpected amplification and improved the percentage of genome recovery from 59% to 89%. Our results demonstrate that microfluidic-generated droplets show potential as an efficient tool for effective amplification of low-input DNA for single-cell genomics and greatly reduce the cost and labor investment required for determination of nearly complete genome sequences of uncultured bacteria from environmental samples.

## Introduction

Single-cell genomics has enabled the investigation of uncultured microorganisms from a broad range of environmental samples [1–5]. Recently, complete or partial genome sequences of uncultured bacteria collected from hot spring sediment [6], a hospital sink [7], sponge symbionts [1], and marine, brackish, freshwater, and hydrothermal samples [8] have been obtained using single-cell sequencing, offering insights into their genetic and metabolic diversity [8, 9]. However, next-generation DNA sequencing (NGS) typically requires nanogram to microgram levels of input DNA. Uncultured microbes isolated from environmental samples naturally contain only a few femtograms of DNA. Thus, whole-genome amplification (WGA) is required to amplify bacterial DNA to adequate quantity without altering the representation of the original DNA sample [10, 11].

Multiple displacement amplification (MDA) [12], using phi29 DNA polymerase and random primers, is the most widely used method for single-cell whole genome amplification. It generates a sufficient quantity of replicated DNA, with high fidelity and large fragment size (10–20 kb), under isothermal reaction conditions. However, several characteristics of MDA raise concerns for obtaining complete genome sequences from small quantities of DNA obtained from uncultured bacteria [4, 13]. First, amplification bias results in differences of orders of magnitude in coverage, and lack of coverage in some regions [14, 15]. Second, formation of genomic rearrangements or chimeras complicates genome assembly by linking non-contiguous genomic regions [16]. Finally, background amplification of contaminating DNA is a major problem. DNA contamination arises from the laboratory environment and the reagents used in the experiments. In fact, contaminant DNA in MDA reagents for a 50-μL-tube reaction is estimated to be on the order of 1 femtogram, equivalent to an entire microbial genome [17]. These problems cause misunderstandings when investigating uncultured microorganisms that lack a reference genome, as non-target sequences can incorrectly be ascribed to the target organism.

To date, many research groups have reported various improvements to MDA methods to overcome these problems. For example, UV treatment of all disposable tubes, plates, and buffers before use has become a common practice in the field of single cell genomics [16, 18]. To further eliminate contaminating nucleic acids, MDA has been performed using stringently decontaminated equipment and buffers in a very clean environment, using ethylene oxide [19] and highly purified Phi29 polymerase prepared in-house [17]. To minimize amplification bias, molecular crowding agents such as trehalose or PEG400 are added to increase the effective template concentration of low-input DNA [20, 21]. As a post-amplification normalization technique, a duplex-specific nuclease has been used to remove high-abundance double-stranded DNA (dsDNA) from amplified MDA products [22]. For clonal cells, the bias can be reduced by pooling of MDA reactions from different individuals [7, 23] or artificially inducing polyploidy, to increase the quantity of clonal DNA from single bacteria [24]. Furthermore, shrinking reaction volumes using microfluidic systems, such as nanoliter-scale chambers, has the effect of concentrating the template with respect to reagent-borne contaminants in proportion to the volume reduction factor [14]. In addition, a recent single-cell assembler, SPAdes improved genome assembly algorithm for dealing with non-uniform coverage and chimeras [25, 26]. Although the above physical and bioinformatic approaches have improved the efficiency of single-cell sequencing, a simpler and more effective method of removing contaminants from the reaction environment and reducing amplification bias has not yet been fully explored.

Recently, microfluidic devices with nanoliter-scale chambers have been widely used for single-cell genetic analyses, including quantitative PCR [27], RNA-seq [28], and WGA [29–31]. Microfluidic devices can integrate labor-intensive experimental processes in a single, closed device and minimize the chance of contamination with exogenous DNA, RNA, DNase, or RNase, which frequently occur in bench-top experimentation. For both DNA and RNA, reaction in microfluidic chambers offers advantages over tube-based approaches, including improved reaction efficiency and detection sensitivity at the single-molecule level [14, 32, 33]. However, the maximum number of reaction compartments is currently ~10^4^ due to the limitations of microfabrication and liquid control in parallel microchambers. Meanwhile, droplet-based microfluidics have also been used for single-cell analysis [34–37] and showed the potential to improve the number and size of compartmentalized reaction environments for DNA and RNA. Microfluidics can generate nano- to femtoliter-sized droplets with high speed and reproducibility by introducing both aqueous solution and immiscible oil. We have demonstrated that picoliter droplets enable high-throughput screening of a metagenomic library constructed from environmental microbes while significantly reducing the cost and time factors [38]. Compartmentalization of cells or nucleic acids in surfactant-stabilized droplets can isolate individual reaction vessels, eliminating the risks of cross-contamination and encounters with reagent-borne contaminants inside the droplets.

Here, we present a novel MDA method to minimize amplification bias and amplification of contaminants using picoliter-sized droplets for compartmentalization of reactions. Single *Escherichia coli* (*E*. *coli*) cells were prepared by Fluorescence Activated Cell Sorting (FACS) and then lysed in tubes. Lysed cell suspensions (10 μL) were converted into approximately 1.5 × 10^5^ droplets (67 pL) within 4 minutes by flow through simple microfluidic channels. Compartmentalized genome fragments can be amplified in the closed droplets without the risk of encounter with environmental or reagent-borne contaminants. Theoretically, the number of contaminating fragments within a commercial reagent could be minimized to < 0.001 fragments per droplet. The reaction of droplets can easily be performed using commercial reagents in off-chip incubation with standard laboratory equipment. The product can easily be recovered by artificial coalescence of whole droplets, purified, and prepared for genome sequencing without any special treatment. Compared to conventional tube-scale MDA methods, this method minimized unexpected amplification and improved the evenness of amplification. Our results demonstrate the potential of microfluidics-generated droplets as a tool for effective amplification of low-input DNA for single-cell genomics by increasing sequencing efficiency with low sequencing effort, thus allowing effective investigation of complete genomes of uncultured microbes collected from environmental samples.

## Materials and Methods

### Bacterial sample preparation

For sequencing analysis of single microbial cells, the *E*. *coli* K-12 strain (ATCC 10798, genome size: 4.6 Mbp) was used as a model, for comparison of amplification properties with previous reports [14, 22, 39]. *E*. *coli* K-12 cells were pre-cultured in Luria-Bertani (LB) medium (1.0% Bacto tryptone, 0.5% yeast extract, 1.0% NaCl, pH 7.0) for 16 h, and collected by centrifugation. The collected cells were washed three times with nuclease-free water (Qiagen, Valencia, CA) with UV treatment. For the preparation of single-cell samples, cells were sorted into 96-well plates using a BD FACS Aria II (BD BioSciences, San Jose, CA) with Syto9 staining, as previously described [1].

### Fabrication of the microfluidic droplet generator

A flow-focusing microfluidic device was designed using AutoCAD (AutoDesk, Sausalito, CA) according to a previously reported design [37], and fabricated using conventional soft lithography techniques. A photomask pattern was transferred to a layer of negative photoresist (SU8-3050, Microchem, Newton, MA) coated on a glass wafer (40 mm × 49 mm), and a master mold was made. All microchannels were 50 μm tall and 100 μm wide, except at the cross-junction area. The cross-junction was designed to be 8.5 μm, 17 μm, or 34 μm wide for the aqueous phase and 34 μm wide for the continuous oil phase. Poly(dimethylsiloxane) (PDMS; Sylgard 184: Dow Corning Corp., Midland, MI) and its cross-linker were mixed thoroughly at a ratio of 10:1 (w/w) and then degassed. The PDMS mixture was poured over the master mold and cured for at least 2 h at 70°C. After curing, the PDMS slabs were carefully peeled off the molds, and the slabs were punched with a 0.75-mm biopsy punch (World Precision Instruments, Sarasota, FL) for connection to syringes via tubes. The punched PDMS slabs and PDMS-coated glass slides ware bonded by plasma treatment (Plasma Cleaner PDG-32G, Harrick Scientific, Ossining, NY), followed by baking for at least 30 min at 70°C. Finally, to produce a hydrophobic surface coating, the microchannel was filled with Aquapel solution (PPG Industries, Pittsburgh, PA), and then excess Aquapel was blown off with air.

### Preparation of MDA mixture for low-input DNA and single bacterial cells

For monitoring of droplet MDA, commercialized lambda DNA (Takara Bio Inc., Shiga, Japan, 48 kbp) was used as a template. To perform an amplification of low-input DNA, lambda DNA was serially diluted with UV-treated nuclease-free water at a concentration of 54 and 265 attogram per droplet and heated at 95°C for 3 min for denaturation. For quantification and sequence analysis of a single-cell genome, *E*.*coli* K12 cells were sorted by FACS into individual reaction tubes containing 1.9 μL of nuclease-free water. Each cell suspension was heated at 95°C for 3 min for cell lysis and DNA denaturation.

For droplet-based MDA reactions, we used a commercially available MDA kit (Genomiphi V2 DNA amplification Kit, GE Healthcare, Waukesha, WI), according to the manufacturer’s protocol, with minor modifications. Prior to reagent introduction into the device, an MDA mixture was prepared containing 2.9 μL of sample buffer, 3.8 μL of reaction buffer, 0.4 μL of enzyme mix, 1 μL of 10% Tween-20 (1% v/v concentration) for use in a 10-μL reaction volume with 1.9 μL of DNA or cell sample solution. For lambda DNA samples, 0.9 μL of nuclease-free water was added to 1 μL of each denatured DNA solution. For monitoring of MDA, 0.5 μL of nuclease-free water was replaced with an equivalent amount of Evagreen (0.5× concentration, Biotium Inc., Hayward, CA). The MDA mixture was mixed gently but completely by vortexing and loaded into the microfluidic device. For comparison with droplet MDA, an in-tube MDA reaction was also prepared according to the manufacturer’s protocol using the same template DNA.

### Droplet MDA operation

**Fig 1:**
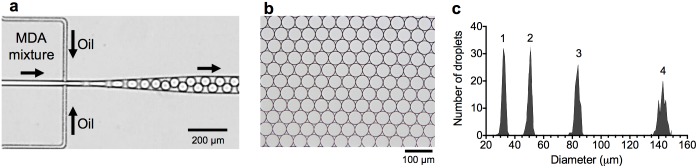
Generation of monodisperse picoliter droplets for compartmentalized MDA reactions. (a) Microphotograph of droplet generation at the microfluidic cross-junction. The MDA mixtures containing single-cell genomes were introduced into the microfluidic device for encapsulation in droplets at the single molecule level. (b) Microphotograph of droplets collected from the microfluidic droplet generator. (c) Size distribution of droplets used for compartmentalized MDA reactions. Ratio of flow rates (water: oil): (1) 2:4, (2) 3:3, (3) 3:1, and (4) 6:1 μL/min. Junction width: (1): 8.5 μm, (2 and 3): 17 μm, and (4): 34 μm.

In our microfluidic device, MDA mixtures containing template DNA or lysed cells were pumped into the cross-junction as a dispersed-phase liquid, while the carrier phase fluorinated oil (HFE7500, Dolomite) containing 2% (v/v) of the surfactant Pico-Surf1 (Dolomite, Charleston, MA) was driven from the other inlet using syringe pumps (KDS 210, KDS Scientific, Hillston, MA). These two phases met at the cross-junction, and droplets were periodically pinched off from the dispersed phase, at a flow rate of 180 μL/h for both the MDA mixture and the carrier oil (Fig 1a). The device outlet was also connected to a collecting PCR tube via PTFE tubing (AWG 24). The 10 μL of MDA mixture was converted into approximately 1.5 × 10^5^ droplets. The extracted DNA fragments were distributed into individual droplets. The collected droplets were incubated at 30°C in PCR tubes using a Veriti ® thermal cycler (Applied Biosystems, Foster City, CA) for 4 h. For comparison, 10-μL in-tube MDA reactions were also conducted at 30°C for 4 h.

### Image analysis

Collected droplets were then transferred into capillary tubes (VitroCom, Mountain Lakes, NJ) for microscopic observation. Bright-field and fluorescent images were captured every 20 min using a fluorescence microscope (BX51; Olympus Corporation) integrated with a digital camera (DP-73; Olympus Corporation, Japan). The diameter of the generated droplets was calculated using ImageJ software (http://rsb.info.nih.gov/ij). The Lumina Vision acquisition software (Mitani Corporation, Tokyo, Japan) was used to analyze the fluorescent images, and the time-dependent changes in the fluorescence intensity of each droplet were analyzed. 100 droplets were analyzed to acquire the average intensity of fluorescent positive droplets at each time point.

### Amplicon quantification

After the MDA reaction, droplets were broken with 1H,1H,2H,2H-perfluoro octanol (Sigma-Aldrich, Poole, UK). The concentration of dsDNA was measured using a Quantifluor minifluorometer (Promega, Madison, WI). For evaluation of copy number biases, we used quantitative PCR (qPCR). We chose ten different single-copy loci from the *E*. *coli* genome, and the copy number of each locus was calculated using Taqman assays [11, 14].

### Library construction and sequencing

For the sequencing analysis, an Illumina library was prepared using amplicons from the droplet MDA and conventional tube MDA. Before library construction, all amplicons were treated with S1 nuclease (Takara Bio Inc., Shiga, Japan) according to the manufacturer’s instructions. After the enzymatic reaction at 25°C for 15 min, 0.5 M EDTA was added to stop the reaction. The reaction mixture was purified using a DNA Clean & Concentrator kit (Zymo Research, Orange, CA). Then, the Illumina library was prepared using all purified amplicons using a Nextera XT DNA sample prep kit (Illumina, San Diego, CA) according to the manufacturer’s instructions. Each library was sequenced on an Illumina Miseq instrument using 2 × 300 paired-end reads.

### Mapping and *de novo* assembly

Acquired reads were normalized to 0.01 to 1 million paired-end reads for each sample. All sequence data were mapped to the NCBI reference genome of NC_00913 (*E*. *coli* substrain MG1655) using the software BWA [40]. Genome coverage was calculated using SAMtools [41]. Each normalized read was assembled de novo using SPAdes 3.5.0 [26], and the contigs were qualified by QUAST 2.3 [42].

### Accession number

The sequence data for single or 10 *E*.*coli* cells amplified with the droplet MDA and single *E*.*coli* cells amplified with the in-tube MDA have been deposited in DNA Data Bank of Japan (DDBJ) under the accession number of DRA003579.

## Results and Discussion

### Genome amplification in a compartmentalized picoliter reaction environment

We designed and fabricated a microfluidic device for generation of picoliter droplets. The format of the device, including the geometry, flow rate, and viscosity, was optimized to generate monodisperse droplets with an average diameter of 50.4 ± 1.3 μm (volume: 67 pL) (Fig 1b). Under these conditions, approximately 700 droplets can be produced per second, resulting in 1.5 × 10^5^ droplets per 10 μL of MDA mixture. Fig 1c shows size distributions of droplets generated by microfluidic device under 4 different conditions. As a result, the droplet sizes were controllable within the range of 30–140 μm (14 pL–1.4 nL) by controlling the flow rate of each phase and the junction width in microfluidic droplet generators (Fig 1c). Thus, the reaction scale of droplets could be easily optimized for improvement of the quality and quantity of MDA products by using microfluidic device. Liquid handling within the microfluidic device requires only one syringe pump and can be performed in a standard experimental laboratory, minimizing the training, time, and labor required.

**Fig 2:**
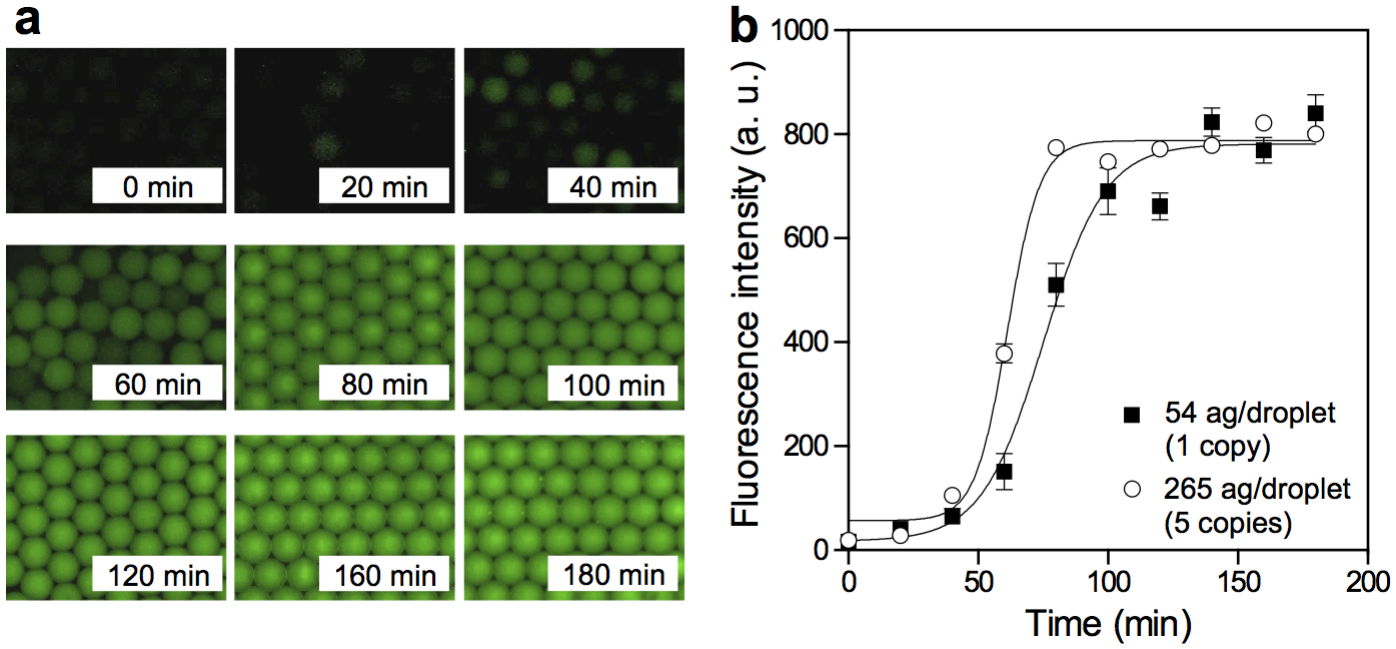
Droplet MDA of low-input lambda DNA. (a) Sequential fluorescent images of droplets encapsulating lambda DNA at a concentration of 265 ag/droplet (5 copies lambda DNA per droplet) with Evagreen dye. (b) Time-dependent appearance of the fluorescence signal during compartmentalized amplification of the denatured lambda DNA (input concentration 54 ag/droplet (1 copy lamda DNA per droplet) and 265 ag/droplet). All data are presented as averaged intensities of fluorescent positive droplets measured with SEM, and 100 droplets were analyzed at each time point.

To validate the amplification workflow, an MDA mixture was emulsified with low-input lambda DNA at the concentration of 54 and 265 attograms/droplet that corresponds to 1 and 5 copies of full length DNA per droplet, respectively. Then, the time-dependent changes in the fluorescence intensities of DNA-intercalating dye in each droplet were monitored. Collected droplets were stably incubated under isothermal MDA reaction at 30°C with the aid of a surfactant. Following incubation, the MDA products were accumulated in individual droplets, resulting in spread of the fluorescent product throughout the droplets (Fig 2a). The time-dependent changes in the fluorescence intensities of the droplets that included 1 copy lambda DNA gradually increased after 60 min and then reached a plateau after 150 min of incubation (Fig 2b). Droplets with higher concentration of lambda DNA showed rapid fluorescence increase compared to droplets with lower concentration of DNA. In addition, their error bars are smaller than those of the droplets with 1 copy lambda DNA because almost all droplets encapsulated several lambda DNA molecules as templates. As a result, the variabilities of fluorescent intensities were small among individual droplets. These results suggest that the proposed microfluidic droplets enabled genome amplification within individual droplets from a single DNA molecule. In addition, a few fluorescent droplets were observed in the no template controls (NTC) droplets, and we consider that the interior fluorescence was due to contaminating DNA fragments. The number of contaminating DNA fragments were calculated from the rate of amplification-positive droplets in NTC samples. From Poisson’s law, the number of contaminating DNA was calculated at the range of 130–492 copies/10 μL (median: 200 copies/10 μL). These values were roughly comparable to the previously reported number of contaminating DNA fragments in the commercial MDA kit (median: 185 copies/10 μL) [17]. Therefore, this system could be applied to validation of reagent lots by evaluating the quantity of contaminant DNA before performing WGA.

### Suppression of unexpected amplification of contaminating DNA

**Fig 3:**
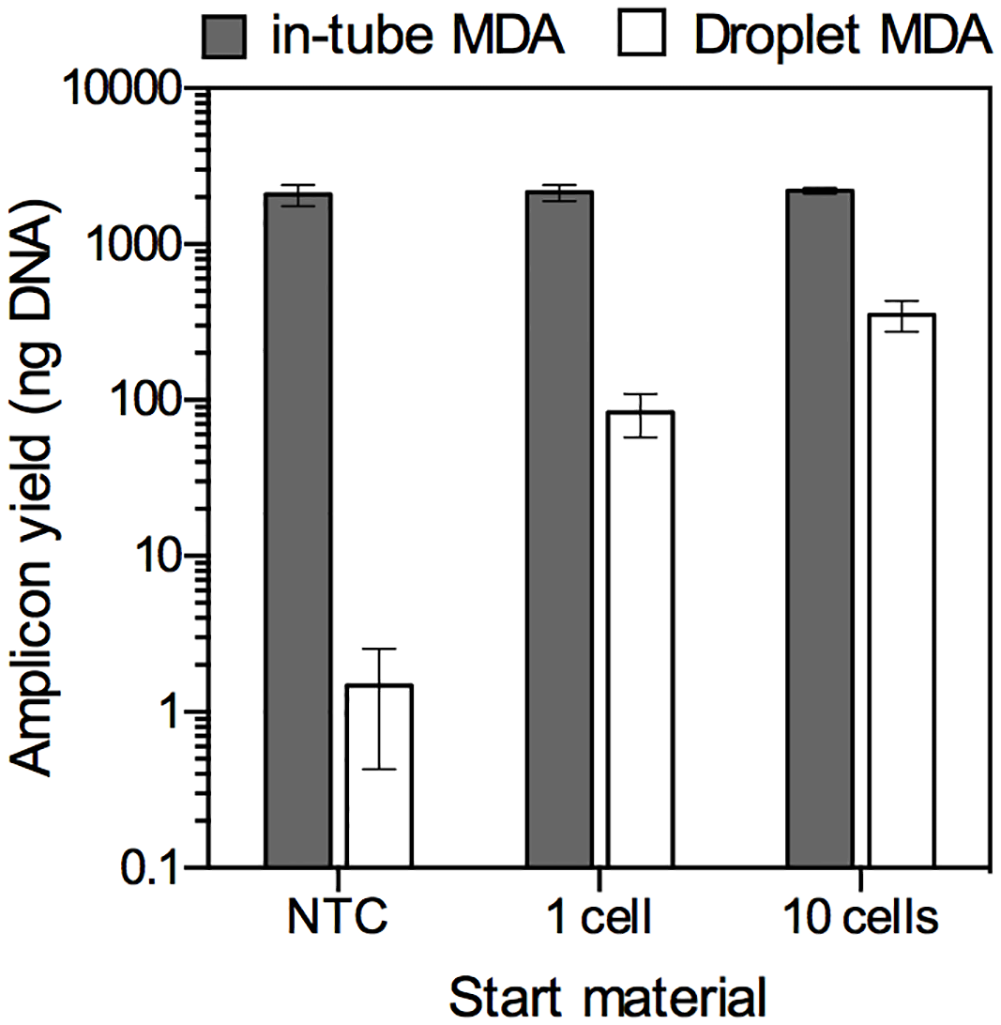
Amplicon yields by in-tube MDA and droplet MDA. No template control (NTC), 1 and 10 *E*. *coli* cells were used as start material. In the droplet MDA, lysed cells were pumped into the droplet generators and genome DNA fragments were randomly encapsulated into picoliter droplets consist of MDA mixture (67 pL per droplet, total 1.5× 10^5^ droplets). After 180 min of MDA reaction, the yields were evaluated following droplet breaking and amplicon purification. A total of 10 μL of MDA mixture was used in both droplet MDA and in-tube MDA reactions.

After the isothermal amplification, we could easily break the emulsified droplets by mixing with perfluorooctanol and recover the MDA products from the aqueous phase. First, we quantified the yield of droplet MDA product for comparison with in-tube MDA product (Fig 3). As control experiments, MDA reactions were performed in a 10-μL volume in tubes, according to the manufacturer’s protocol, with each reaction receiving either a single or 10 *E*. *coli* cells isolated by FACS. Under conventional in-tube conditions, a consistent yield of MDA product (approximately 2.1 μg) was obtained regardless of template quantity. In contrast, the droplet MDA products appeared to be proportional to template quantity. For example, 1.4 ng, 47 ng, and 350 ng of DNA were obtained from samples containing NTC, 1, and 10 cells, respectively. From yield calculations, single-cell genomes were amplified >10^6^-fold by droplet MDA, sufficient for library construction for next-generation sequencing. Remarkably, in droplet MDA, the yield of NTC samples was 1400-fold lower than that of in-tube MDA. In droplet MDA, due to compartmentalization of each DNA fragment in an individual droplet, excess amplification of DNA fragment contaminants could be prevented. The reaction volume is restricted to 67 pL, theoretically resulting in 13–23 pg of amplified DNA in individual droplets, even though one or more DNA molecules are present in each droplet. In fact, the product yield per amplification-positive droplet was calculated to be 7.5–15 pg in the NTC sample. Then, the nature of the MDA product obtained from the NTC samples was determined by assembling contigs (≥500 bp) from all reads and using the BLAST search algorithm for identification. Table 1 shows that the number and total length of the contigs produced by in-tube MDA (584 contigs, 1 Mbp) was much higher than those produced by droplet MDA (34 contigs, 68 kbp). In both cases, contigs from the NTC sample were ascribed to *Homo sapiens*, *Acidoborax*, and *Pseudomonas*, which are often observed as contaminants [18, 32]. These results suggested that the compartmentalized reaction could suppress the unexpected amplification of contaminating DNA fragments by encapsulation in individual closed droplets. Using the droplets as the MDA reaction environment, we could eliminate unnecessary genomic information due to unexpected amplification that can lead to misunderstanding of sample characteristics.

**Table 1:** Assembly statistics of sequence reads obtained from MDA products of contaminants in no template control (NTC) samples.

	Droplet MDA	in-tube MDA
# contigs (≥500 bp)	34	584
Largest contig (bp)	17151	27943
Total length (bp)	68448	1064672
N50 (bp)	5311	3356

A total of 10 μL of MDA mixture was used in both droplet MDA and in-tube MDA reactions. In both droplet MDA and in-tube MDA, a total of 1 ng of MDA product was used for sequence library preparation. Row sequence reads were obtained at 100× sequencing effort.

### Prevention of amplification bias across the genome

To evaluate the effect of bias suppression in compartmentalized reactions, we first compared the abundance of ten loci distributed across the entire *E*. *coli* genome, which were analyzed by qPCR in a previous report [11, 14]. In this assay, amplification bias is indicated by the over- and underrepresentation of the ten loci, which are originally present at one copy per genome. In accordance with the previous report, the amplification bias was far greater for the in-tube MDA reactions (S1 Fig). The average copy number of the ten loci was much higher for droplet MDA (1.2 × 10^5^ copies/ng (product DNA)) than for in-tube MDA reactions from a single *E*. *coli* cell (1.2 × 10^3^ copies/ng (product DNA)). These results indicated that amplification bias between loci was suppressed by droplet MDA, compared to conventional in-tube and nanoliter chamber reactions [14]. In a similar manner to this, emulsion PCR significantly decreases amplification bias because of isolating heterogeneous DNA fragments within individual droplets, resulting prevention of competition between multiple amplicons [43, 44]. Thus, the compartmentalization of heterogeneous DNA fragments into picoliter droplets could significantly prevent the competition and chimeric formation in each fragment in genome amplification. In addition, it implied that the in-tube MDA products contain more unexpected amplicons, derived from contaminating DNA fragments, than the droplet MDA products.

**Fig 4:**
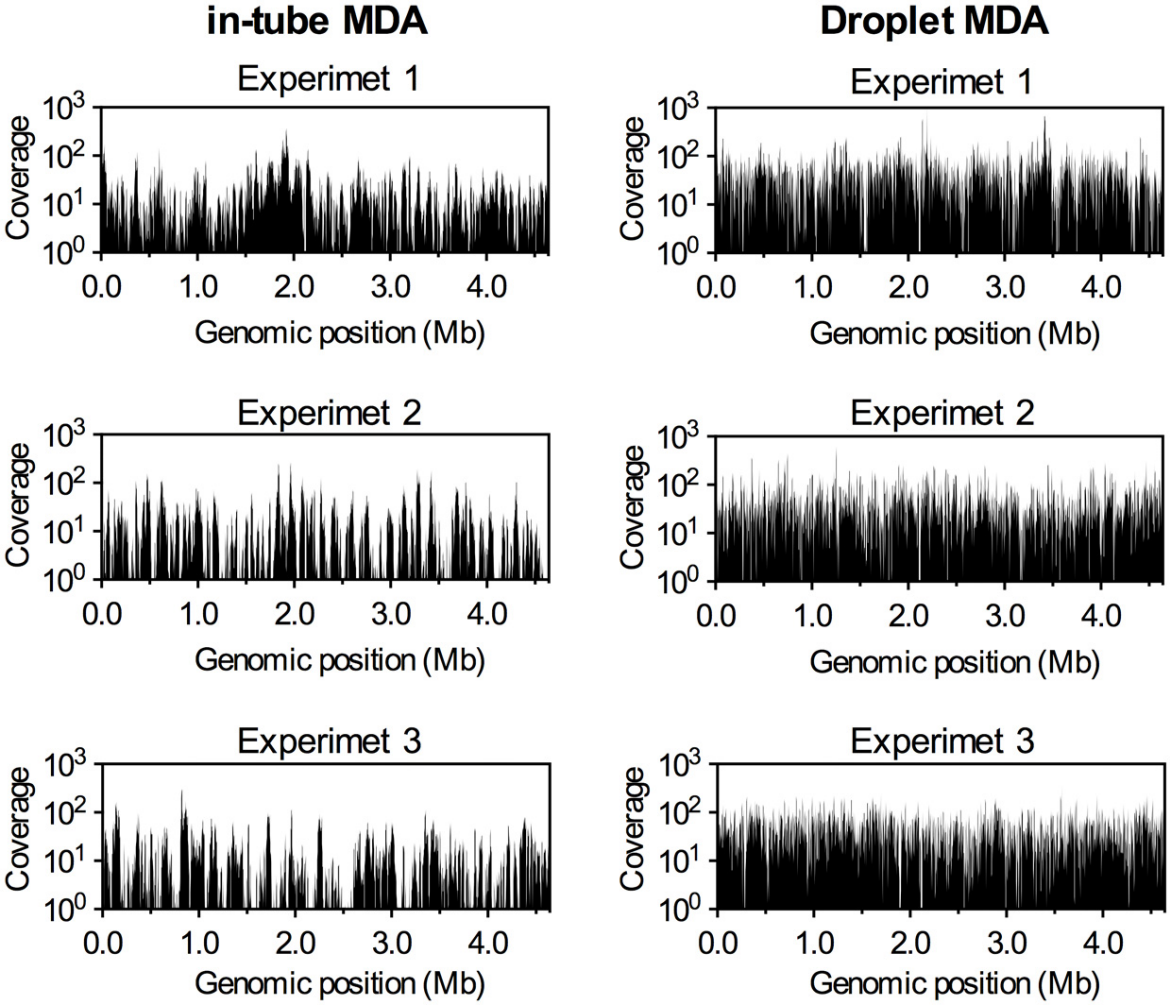
Evaluation of amplification bias of droplet MDA. Distributions of sequencing coverage of MDA products from single *Escherichia coli* cells (n = 3) were compared between in-tube MDA (left column) and droplet MDA (right column). Each graph shows the results of independent reactions. The averaged sequencing coverages were calculated from raw sequencing reads that mapped to with *E*. *coli* reference genome within 1-kb windows. Sequencing reads were normalized to 60× sequencing effort in each experiment.

**Fig 5:**
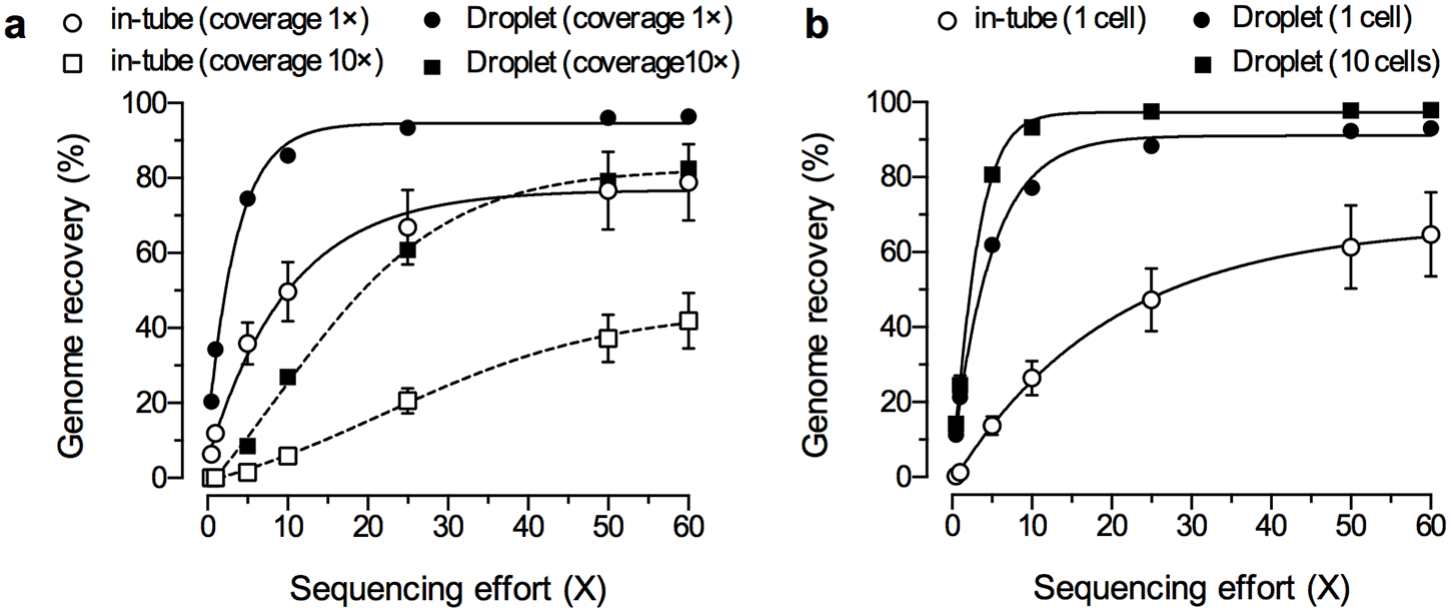
Genome recovery from row sequence reads and *de novo* assembled contigs obtained from droplet MDA products of single and 10 *E*. *coli* cells. (a) Comparison of genome recovery from raw sequence reads as a function of sequencing effort. Each plot shows the averaged percentage of genome recovery with SD from raw sequence reads for single (n = 3) and 10 (n = 3) *Escherichia coli* cells at >1× or >10× sequencing coverage. (b) Comparison of genome recovery from *de novo* assembled contigs as a function of sequencing effort. Each plot shows *de novo* assembly result in in-tube MDA and the droplet MDA.

To further quantify the amplification bias among whole genomes, we generated 1.1 to 2.6 million paired-end Illumina MiSeq sequencing reads, 300 bp in length, for the same MDA products. To evaluate the profiles of sequence reads mapped to the reference genome, the sequencing efforts was normalized to 60×, which means 60 times the amount of sequenced base length to *E*. *coli* genome. Then, the sequencing coverages, which means the number of reads mapped to reference, were calculated among the whole genome from normalized sequence reads by using software BWA. Fig 4 shows sequencing coverage versus genomic position measured for single-cell and 10-cell MDA products. To evaluate the variation among each MDA product, three independent experiments were compared between in-tube and droplet MDA products. Concordant with the qPCR results, all MDA samples displayed a bias in sequencing coverage. In particular, as shown in the histograms in Fig 4, in-tube MDA products of single cells displayed a number of unmapped areas and quite large variations in sequencing coverage among genome position as compared to droplet MDA products. As expected, droplet MDA reduced coverage variation (average coverage: 48 ± 48) compared to in-tube MDA products (average coverage: 17 ± 25), resulting in a more even distribution of the sequencing coverage (Fig 4 and S2 Fig). In particular, the coverage of droplet MDA, using 10 cells, improved the variation (average coverage: 51 ± 20) compared to single-cell MDA products (S3 Fig). Thus, a sufficient quantity of clonal cells could improve the reproducibility of MDA and provide balanced sequencing coverage. A comparison of the percentage of the genome recovery versus the sequencing effort revealed that the genome recovery of droplet MDA was higher than that of in-tube MDA (Fig 5a). For example, when the sequencing effort was 60×, 83% and 42% of the genome was recovered from single cells at >10× sequencing coverage by droplet and in-tube MDA, respectively. The steep increase of the curve for droplet MDA indicated its minimal amplification bias, resulting in low variation in coverage across the genome. In addition, the inter-reaction variation of droplet MDA was less than that of in-tube MDA, resulting in small error bars in Fig 5. Previous studies have reported that the genome recovery rate from single *E*. *coli* cells was 40–67% in the in-tube MDA [14, 32]. These rates are comparable to our in-tube MDA results. Moreover, MDA in nanoliter chambers has recovered 30–50% more of the *E*. *coli* genome.by the effect of reducing the reaction volume to reduce contamination and amplification bias [14, 29]. Although sequencing methods and analysis algorithm are different from each other, our picoliter droplets also demonstrated high genome recovery rate with low sequencing effort.

### *De novo* assembly from droplet MDA product

*De novo* assembly of the genome was then performed using SPAdes [25, 26] and the quality of assembled sequence reads was evaluated using QUAST [42]. From the droplet MDA products, the assembled contigs recovered 88–91% of the *E*. *coli* genome from single cells, and consistently recovered 98% of the genome from 10 cells, at 60× sequencing effort (Fig 5b). This means that even in the *de novo* assembly, nearly complete *E*. *coli* genome was obtained in the droplet MDA. In addition, the total length of contigs unaligned to reference genome was 0.77 Mbp in the droplet MDA, while it was 3.4 Mbp in the in-tube MDA. This suggested that in-tube MDA products contained a large quantity of unexpected amplicons derived from DNA contaminants. In the in-tube MDA, excess of unexpected amplicons spoiled the quality of contigs, resulting in the increase in contig number and total contig length (Table 2). In comparison, droplet MDA generated a small number of contigs with a higher N50 value, which is the median length of all contigs. When the starting material was increased to 10 cells, the number of contigs and N50 of droplet MDA were further improved, although the total contig lengths were comparable. In terms of structural errors in the contigs, droplet MDA clearly reduced the ratio of misassembled contigs, mismatches, and indels per 100 kbp relative to in-tube MDA. These results indicated that droplet MDA could decrease the number of unexpected contigs due to contaminants, the occurrence of chimeric fragments, and misassembly between target and contaminants. Therefore, we consider that droplet MDA could provide qualified genome assembly from a single-cell because of compartmentalized amplification of target DNA and contaminants in uniformed reaction vessels.

**Table 2:** Assembly statistics of MDA products obtained from single *Escherichia coli* cells.

	1 cell in-tube MDA (n = 3)	1 cell droplet MDA (n = 3)	10 cells droplet MDA (n = 3)
# contigs (≥500 bp)	3045 ± 313	1400 ± 243	136 ± 24
Total length (kbp)	5784 ± 414	4833 ± 50	4688 ± 25
N50 (bp)	3644 ± 210	11287 ± 2757	123806 ± 7588
**Statistics with reference genome**			
# misassembled contigs	106 ± 24	34 ± 3	22 ± 1
# fully unaligned contigs	1988 ± 323	500 ± 120	25 ± 25
# partially unaligned contigs	279 ± 68	146 ± 10	2 ± 2
# mismatches per 100 kbp	35 ± 2	17 ± 2	4 ± 0.3
# indels per 100 kbp	2.3 ± 0.5	1.1 ± 0.1	0.2 ± 0
Genome fraction (%)	59 ± 11	89 ± 2	98 ± 0

A total of 10 μL of MDA product was evaluated for both droplet MDA and in-tube MDA. Sequencing reads were normalized to 0.8 M reads (60× sequencing effort) in each experiment.

The contigs obtained from droplet MDA using 10 cells recovered a much larger fraction of the genome than conventional in-tube MDA, at low sequencing effort (<25×). This result demonstrates that droplet MDA provides a much more efficient way to assemble whole bacterial genomes from a small population of clonal cells. In previous reports, gel microdroplets were used for growth of genetically identical cells, as an input for MDA [34, 39]. As we demonstrated [38], droplet technology facilitates handling of single bacterial cells in compartmentalized environments. Increasing the cell input is a simple yet efficient way to improve the quality of amplicon and obtain qualified sequence reads. Thus, we consider that the combination of droplet MDA with small clonal cell populations [24, 34, 39] and/or a mini-metagenomic approach [7] would be useful for obtaining near-complete genome sequences with minimum sequencing effort. We believed that droplets show great potential as a platform for implementation of entire processes, including isolation of single cells, culturing multiple clonal cells from single cells, low-bias and contamination-free MDA, and recovery of complete genomes of environmental microbes.

## Conclusions

Droplets can provide a low-bias and contamination-free WGA environment, and improve the genome coverage of MDA products from single cells. We demonstrated that droplet MDA has the potential to produce high-quality genomic data from single cells with low sequencing effort. In addition, this method could play an important role in quality control of reagent lots by digital detection of contaminating DNA. It could be useful in the exploration of low-abundance diversity in mini-metagenomes and metatranscriptomics by reducing amplification bias. We believe that this technique has the potential for extending our understanding of microbial genomic diversity.

## Supporting Information

S1 Fig qPCR analysis for evaluation of the representation of 10 loci in single-cell MDA products. FACS-sorted single *Escherichia coli* cells (n = 3) were lysed and their gDNA was amplified using droplet MDA and in-tube MDA. The gene copy numbers in each MDA product were estimated by qPCR. In both droplet MDA and in-tube MDA, a total of 10 μL of MDA product was prepared. Each bar represents an individual sample set.
(TIF)

S2 Fig Histograms of sequencing coverages of droplet MDA and in-tube MDA products. The x-axis shows the log10 ratio of sequencing coverages. The averaged sequencing coverages were calculated from raw sequencing reads that mapped to with *E*. *coli* reference genome within 1-kb windows. Sequencing reads were normalized to 60× sequencing effort in each experiment.
(TIFF)

S3 Fig Evaluation of amplification bias of droplet MDA from 10 cells. Distributions of sequencing coverage of MDA products from 10 *Escherichia coli* cells (n = 3) prepared by droplet MDA in a total reaction volume of 10 μL. Each graph shows the results of independent reactions. Genomic positions were consolidated into 1-kb windows. The averaged sequencing coverages were calculated from raw sequencing reads that mapped to with *E*. *coli* reference genome within 1-kb windows. Sequencing reads were normalized to 60× sequencing effort in each experiment.
(TIF)
