# Supplementary figures and images for: Monodisperse Picoliter Droplets for Low-Bias and Contamination-Free Reactions in Single-Cell Whole Genome Amplification
Source: PLoS One. 2015 Sep 21;10(9):e0138733. doi: 10.1371/journal.pone.0138733 (PMC4577099; doi:10.1371/journal.pone.0138733)

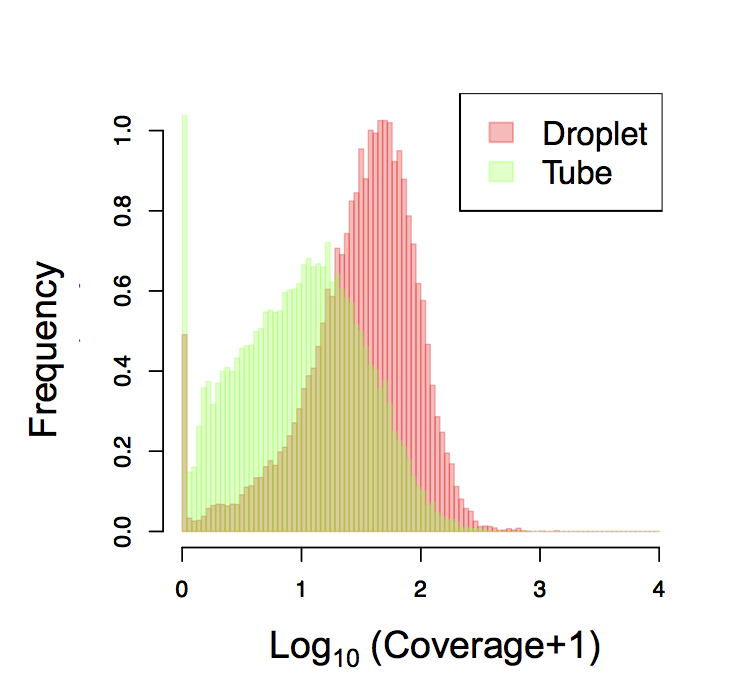

Supplement: S2 Fig — The x-axis shows the log10 ratio of sequencing coverages. The averaged sequencing coverages were calculated from raw sequencing reads that mapped to with E. coli reference genome within 1-kb windows. Sequencing reads were normalized to 60× sequencing effort in each experiment. (TIFF) [file pone.0138733.s002.tiff]
